# How can the pain sensitivity to be affected by maximal progressive exercise test during pregnancy?

**DOI:** 10.1371/journal.pone.0300058

**Published:** 2024-08-07

**Authors:** Katarzyna Leźnicka, Agata Gasiorowska, Maciej Pawlak, Aleksandra Jażdżewska, Agnieszka Maciejewska-Skrendo, Anna Lubkowska, Anna Szumilewicz

**Affiliations:** 1 Faculty of Physical Culture, Gdansk University of Physical Education and Sport, Gdansk, Poland; 2 Faculty of Psychology in Wroclaw, SWPS University of Social Sciences and Humanities, Ostrowskiego, Wroclaw; 3 Department of Physiology and Biochemistry, Poznan University of Physical Education, Królowej Jadwigi, Poznań, Poland; 4 Institute of Physical Culture Sciences University of Szczecin, Szczecin, Poland; 5 Department of Functional Diagnostics and Physical Medicine, Faculty of Health Sciences, Pomeranian Medical University, Szczecin, Poland; Medical University of Graz: Medizinische Universitat Graz, AUSTRIA

## Abstract

The multidimensional etiology of pain may explain the beneficial effects of regular physical activity, as evidenced by increased pain tolerance. Physically active people find it easier to exert themselves, which enables them to increase their physical activity, which in turn leads to a reduction in pain. However, no study investigated the physical activity and exercise tests as modulators of pain sensitivity in pregnant women. Therefore, this study aimed to investigate the changes in pain perception in pregnant women during pregnancy, with a particular interest in the effects of maximal progressive exercise test (CPET) and self-performed physical activity (PA). Thirty-one women with an uncomplicated singleton pregnancy (aged 23–41 years; M = 31.29, SD = 4.18) were invited to participate in pain sensitivity measurements before and after CPET twice during pregnancy (with an 8-week break). We found that pregnant women had a significantly lower pain threshold after a maximal exercise test than before, regardless of whether the test was performed in the second or third trimester of pregnancy. This effect was most pronounced in women with low levels of physical activity. Second, women with high physical activity had higher pain tolerance than women with moderate and low physical activity. In addition, physical activity levels predicted changes in pain tolerance over the course of pregnancy, with negative changes in women with low physical activity and positive changes in women with moderate physical activity. Finally, these associations were not reflected in differences in the subjective pain experience.

## Introduction

Pain is primarily a subjective feeling and one of the most powerful stressors for the human body. A modified definition published by the International Association for the Study of Pain (IASP) [1]) describes this phenomenon more comprehensively as “An unpleasant sensory and emotional experience associated with, or resembling that associated with, actual or potential tissue damage.” Although the phenomenon of pain has been thoroughly researched from structural and functional aspects, some questions remain about pain measurement in clinical applications. First and foremost, human pain perception is characterized by high individual variability, which is primarily based on genetic or environmental factors [2], but can also be influenced by psychological, social, cultural, and even religious or spiritual factors. In other words, the intensity and mode of pain experience, as well as the ability to control pain, are influenced by various factors, including location and duration of pain, individual patient characteristics such as personality or temperament, previous pain experiences, life satisfaction, social contacts, physical activity (PA), etc. All of these predictors "determine" whether the same noxious stimulus is perceived as more or less painful [3].

The multidimensional etiology of pain may explain the positive effects of regular PA, which are manifested in increased pain tolerance [4, 5]. Physically active individuals find it easier to exert themselves, which allows them to increase their PA, which in turn leads to a reduction in pain [6]. Previous research suggests that even relatively low levels of physical exertion can modulate the perceived threshold of painful and non-painful stimuli in short term outcomes [7, 8]. This is confirmed by the conclusions of the meta-analysis [9] and a separate study among martial artists [10]. The authors found higher pain tolerance in athletes compared to the control group, which consisted of non-exercising individuals, and indicated that regular PA is associated with changes in pain perception, although conclusions were unclear in the case of pain threshold. Such an effect, termed "acute exercise-induced hypoalgesia," lasts for a short and variable period of time, typically less than 30 minutes after a single exercise session [9]. Furthermore, high levels of physical activity correlate with greater conditioned pain modulation (CPM) in healthy control subjects [11] CPM characterize the capabilities of the pain system, particularly the central modulation of pain, which is higher in athletes [12] and predicts exercise-induced analgesia in healthy individuals [13, 14]. The beneficial effects of PA appear to be particularly important for childbirth, when the effect of "acute exercise-induced hypoalgesia" may be especially beneficial. However, to the best of our knowledge, no study has examined physical activity and exercise as modulators of pain sensitivity in pregnant women.

Currently, pregnant women are advised to use PA as a necessary condition for a good pregnancy and puerperium [15]. Major benefits of PA during pregnancy include reduced complications, injuries, musculoskeletal trauma, and maternal harm during delivery [16]. Although there are some studies in which authors have investigated pain perception in response to pressure pain (mechanical stimulation) or cold using the Cold Pressor Test (CPT) [17, 18], most of them have mainly focused on analyzing specific factors affecting only postpartum pain, making it difficult to relate them to general pain sensitivity [19]. We, therefore, see the need to systematically analyze the relationship between physical activity and our variables of interest.

Thus, the aim of this study was to investigate changes in pain perception in pregnant women during pregnancy, with particular interest in the effects of the maximal progressive stress test and self-performed PA on pain experience. In addition to measuring subjective pain perception, participants underwent more objective physiological tests that measured pain threshold and pressure pain tolerance using an algometer. These measurements were taken before and after a maximal progressive stress test at two different time points during pregnancy, with a time span of approximately 8 weeks.

## Materials and methods

### 1. Participants

Thirty-one pregnant women in singleton, uncomplicated pregnancy, were recruited to participate in the eight-week, online educational program on healthy lifestyle and physical activity (aged 23–41; *M* = 31.29, *SD* = 4.18). Participants recruitment began on April 26, 2021 and ended on April 30, 2021. Most participants (*n* = 26, 83.87%) did not give birth before. The measurements took place two times, with the first measurement (T1) on average in the 23.81 weeks of pregnancy (*SD* = 3.97, range 13–28) and the second measurement (T2) in the 32.85 weeks of pregnancy (*SD* = 3.91, range 22–40). Eleven women did not complete the program and did not take part in the second measurement for the following reasons: the lack of doctor’s permission to further participate in the study (*n* = 2), not being interested in continuing the program (*n* = 4), preterm birth not related with the lifestyle factors or intervention (*n* = 1), taking medications that could influence other study outcomes (*n* = 1); not feeling well on the day of the second assessment (*n* = 2), or did not provide a reason (*n* = 1). Due to the high turnover, an attrition analysis was performed before the main analysis to test whether the final sample did not differ from the initial sample concerning the levels of the variables of interest, hence allowing for generalization.

### 2. Procedure

During the recruitment phase, demographic data and informed consent were obtained from study participants. In addition, their physical activity level was measured using the short version of the International Physical Activity Questionnaire (IPAQ) (20. Then, participants were invited to the laboratory of physical effort for measurements at time 1 (T1). First, objective pain experience, operationalized as pain threshold (PPT) and pain tolerance (PTOL), was measured with an Algometer, and subjective pain experience was measured with the Visual Analog Scale (VAS). After these measurements, the women underwent a progressive cardiopulmonary exercise test (CPET), followed by a 3-minute rest period. Thereafter, the assessment of pain perception PPT, PTOL, and VAS was performed again.

After completing an 8-week educational program, all participants completed the IPAQ and were asked to return to the designated room in the laboratory of physical effort for measurements at time 2 (T2), the protocol of which was the same as at T1. Participants underwent measurements of PPT, PTOL, and subjective pain perception, followed by CPET with 3 minutes of rest and another series of measurements of PPT, PTOL, and subjective pain perception.

#### 2.1 Physical activity

**Fig 1:**
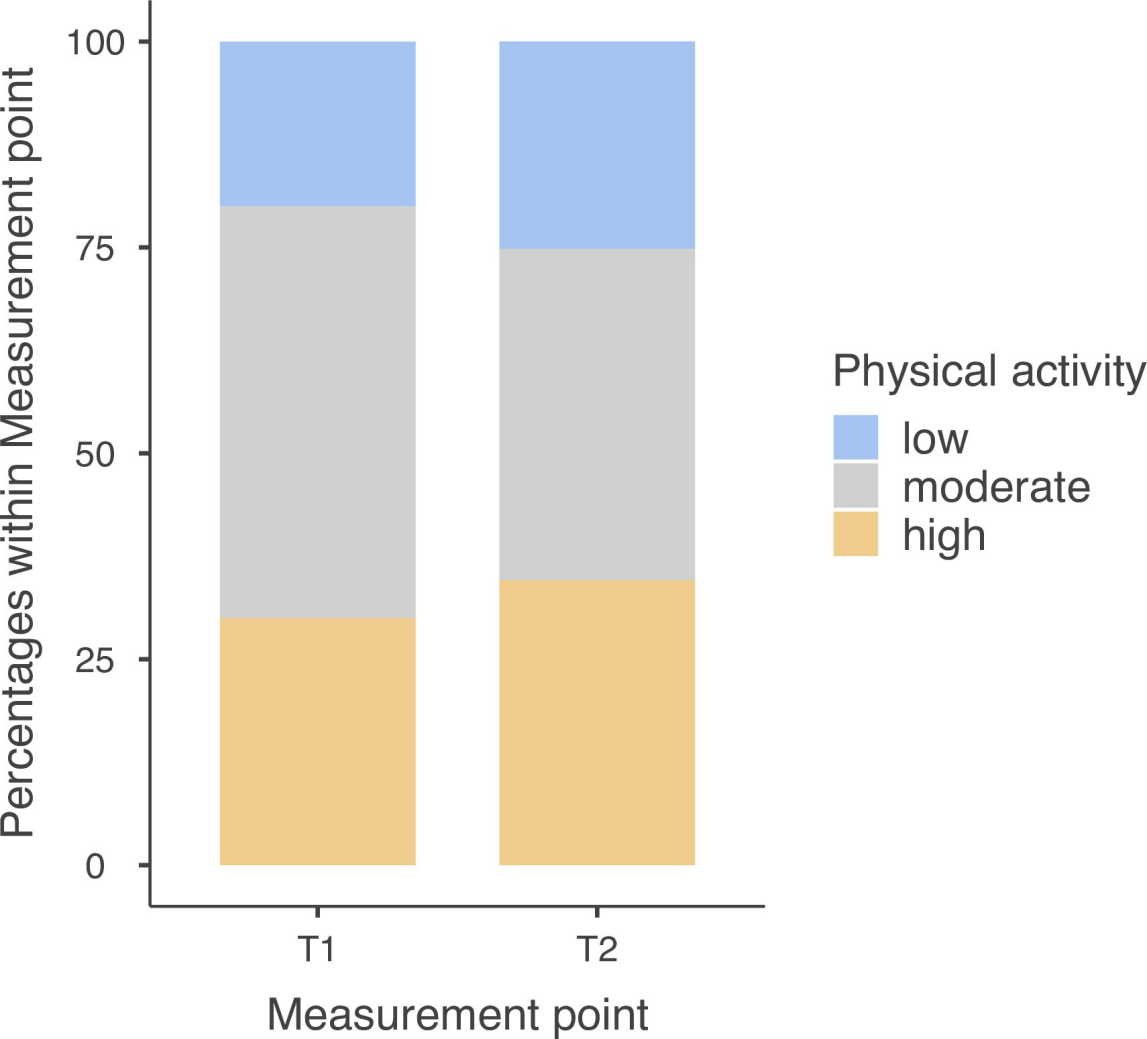
The levels of participants’ physical activity as measured with IPAQ at T1 and T2.

The level of physical activity in pregnant women was measured using the short form of the International Physical Activity Questionnaire (20. This questionnaire has satisfactory reliability and validity and provides information on weekly physical activity levels in multiples of resting metabolic rate (MET). Based on the IPAQ results, pregnant women were classified into three levels (categories) of PA: low (inactive women), moderate (reaching a minimum recommended level of PA), and high (exceeding the minimum recommended level of PA) [20]. The proportion of women classified into the three levels did not differ between the two measurement time points T1 and T2, χ2(2, N = 20) = 2.01, p = 0.735 (see Fig 1). Almost half of the participants did not change their PA level (n = 9). Six participants reported increasing their PA level (*n* = 6), while five others reported decreasing their PA level (*n* = 5). The level of PA was negatively associated with gestational week, Spearman’s ρ = -.12, p = .034.

#### 2.2 Pain measurement

PPT, PTOL. Tissue pressure sensitivity measurements were performed using an Algometer from Medoc AlgoMed (Israel). The AlgoMed system consists of a digital head connected to a computer that can perform various pressure test protocols at selected points on the body. The researcher places the digital head at the test site and presses on the tissue at an increasing rate (kPa/s). The result is visible in the head window and is stored in the corresponding file. Pain threshold (PPT) is defined as the minimum amount of pressure required for the sensation of pressure to first change to pain, whereas pressure pain tolerance (PTOL) describes the maximum stimulus intensity or duration of continuous painful stimulation that a person is willing to endure.

The participants were informed about the functioning of the device and then a test measurement was performed. They were tested in a sitting position, and measurements were taken on both upper limbs on the dorsum of the hand between the thumb and index finger and on the lateral surface of the arm at 2/3 of the shaft length. All measurements were taken by the same researcher, and always in the morning. The investigator placed the algometer head on the area to be examined and gradually applied stimuli at a rate of 30 kPa/sec. When pain occurred, the participant said "stop." This measurement was used as an indicator of pain threshold (PPT result). The measurement was then continued until the participant could no longer tolerate the stimulus and signaled the end of the measurement. The point at which a painful pressure stimulus could no longer be tolerated was used as the pain tolerance measurement (PTOL result).

#### 2.3 Visual analog scale (VAS)

After completion of the PPT and PTOL tests, participants were asked to report their subjective pain intensity using the Visual Analog Scale (VAS) to assess the extent of subjective pain during the procedure compared with their own subjective perception of pain. Pain intensity was rated on a scale from 0 = "no pain and discomfort" to 10 = "the worst possible pain and discomfort."

#### 2.3 The cardiopulmonary exercise test (CPET)

The maximal progressive cardiopulmonary exercise test (CPET) was conducted according to recommendations by the American Thoracic Society/American College of Chest Physicians [4], using a cycle ergometer with an electronically regulated load (Viasprint 150P; Bitz, Germany)

The women began to warm up by cycling for 4 min with a relative load of 0.4 W⸱kg−1 of body mass. When the participants had warmed up, the load was increased by 0.2 W⸱kg−1 per minute until they refused. In preparation for the test, the women were encouraged to cycle up to the limit of their physical capacity. They were also informed that they could stop the test at any time. The participants rested for 3 min after they finished cycling.

#### 2.4 The educational intervention

The educational program focused on a healthy lifestyle and physical activity during pregnancy, according to current recommendations [21, 22] and specific pregnancy and parenting issues. Online classes were held once a week, for one hour for a total of 8 weeks. We encouraged the women to be physically active on their own to achieve at least the minimum recommended level of physical activity, which was at least 150 minutes of moderate to vigorous physical activity per week. We asked the group to record a log of all their PA including daily activities (i.e., cleaning the house, shopping, or gardening) that lasted at least 10 min and any structured exercise sessions. The Talk Test and Borg RPE Scale were employed to measure PA intensity. We suggested a level of exercise intensity at which the study women had a noticeable increase in breathing frequency. We asked them to complete the individual Exercise Monitoring Cards after each session of PA. It contained the following information: date of PA session, form of PA (e.g., walking, cycling, gardening), the duration of PA, subjective assessment of PA intensity at 0–10 RPE scale, rest time after PA session, well-being during or after PA. The Exercise Monitoring Cards was presented by one of the coauthors of this study in the previous work [23]. Women reported that most often they performed physical activity with moderate intensity, on average of 6 ± 1 on the 0–10 Borg’s scale [24].

#### 2.5 Ethics statement

The study was approved by Bioethics Committee of the Regional Medical Chamber in Gdansk (Poland) and numbered KB -8/21. The investigation protocols were conducted ethically according to the World Medical Association Declaration of Helsinki. The participants were informed about the purpose of the study and gave written informed consent to participate in the research. All personal information and results were anonymous and were processed and stored following current regulations of data protection in Poland. This study is a part of a clinical trial registered in ClinicalTrials.gov (NCT05009433).

#### 2.6 Statistical analysis

The data analysis used JASP (descriptive statistics and attrition analysis) and JAMOVI (mixed linear regression) [25–27]. The threshold for statistical significance was set at *p* < 0.05. The distributions of some pain measurement variables significantly differed from a normal distribution (*p*s < .001) such that they were all heavily right-skewed. Hence, the t-test with Welch correction was used for a two-groups comparison (attrition analysis). For the remaining analyses, the data were considered nested, as all participants were subject to pain measurements several times. Hence, multi-level modeling with JAMOVI with REML estimation, allowing for using variables that deviate from the normal distribution, was applied for data analysis.

The mixed-model regressions included the following independent variables: [1] week of the pregnancy at the moment of measurement; [2] level of physical activity declared at the moment of measurement; [3] whether the pain-related variables were measured before or after the exercise test. The regression also included the interactions between the abovementioned variables, controlled whether the measurements were conducted on the hand vs limb and the dominant vs non-dominant hand, and included a random intercept for participants. The regression analysis was conducted three times for the following dependent variables: [1] pain threshold, [2] pain tolerance, and [3] subjective feeling of pain.

The sensitivity analysis using G*Power revealed that with power 1- β = 80% and significance α = 0.05, a sample of *n* = 20 and 16 measurements from each participant correlated on average at .40 is large enough to detect an effect of β = 0.25 in within-group comparisons.

## Results

### Attrition analysis

Table 1 includes the results of a comparison between participants who finished the intervention program vs those who did not complete the program and hence did not participate in the measurements at Time 2, regarding basic sociodemographic features and pain measurement. The results demonstrated that participants who dropped out did not differ from those who completed the intervention regarding age, week of pregnancy, BMI, pain tolerance, and subjective feeling of pain. We observed significant differences regarding participants’ pain threshold, such as those with a higher pain threshold were less likely to drop out of the study. Additionally, the two groups of participants did not differ concerning their dominant hand, χ^2^(1, *N* = 31) = 0.20, p = 0.657, and the declared level of PA before starting the study χ^2^ (2, *N* = 31) = 0.64, *p* = 0.726, with most of the participants declaring medium activity (*n* = 16, 51.6%) followed by high activity (*n* = 6, 32.3%), with only five participants (16.1%) who indicated low PA before the first measurement time.

**Table 1:** Comparison of the pregnant participants who finished the intervention vs those who dropped out.

Variable	Not participated in the T2 measurement *n* = 11		Participated in the T2 measurement *n* = 20		*t*	df	*p*	Cohen’s d
	*M* ± *SD*		*M* ± *SD*					
Age	29.73	3.98	32.15	4.13	-1.60	21.43	0.124	-0.60
WP	24.09	4.54	23.65	3.67	0.27	17.03	0.789	0.11
BMI	26.11	5.96	25.38	3.18	0.38	13.21	0.710	0.15
PPT dom arm	64.76	37.16	110.28	59.37	**-2.62**	**28.37**	**0.014**	**-0.92**
PPT ndom arm	64.60	20.15	114.17	73.30	**-2.84**	**23.73**	**0.009**	**-0.92**
PPT dom hand	72.26	40.42	115.98	75.64	**-2.10**	**29.00**	**0.045**	**-0.72**
PPT ndom hand	124.65	63.66	129.85	80.94	-0.20	25.20	0.845	-0.07
PTOL dom arm	507.56	190.87	650.18	196.53	-1.97	21.26	0.062	-0.74
PTOL ndom arm	521.88	196.87	576.90	164.47	-0.79	17.77	0.441	-0.30
PTOL dom hand	711.92	220.49	769.18	262.07	-0.65	23.96	0.524	-0.24
PTOL ndom hand	597.67	165.25	719.79	266.77	-1.57	28.45	0.127	-0.55
VAS dom arm	6.64	1.21	6.45	1.19	0.41	20.50	0.684	0.16
VAS ndom arm	7.00	0.63	7.00	1.03	0.01	28.49	1.000	< 0.01
VAS dom hand	6.64	1.75	6.45	1.15	0.32	14.85	0.755	0.13
VAS ndom hand	7.36	0.81	7.15	1.14	0.61	26.85	0.549	0.22

Note: All variables were measured at T1. Pain sensitivity variables were measured before the exercise test. PPT = pain threshold, PTOL = pain tolerance, WP = week of pregnancy. The data are presented as means and standard deviation. Values in bold represent statistically significant differences in the Welch’s t-test at *p* < 0.05

### Pain threshold

The results of multi-level regression conducted with JAMOVI (see Table 2) showed that the independent variables and covariates accounted for R^2^_marginal_ = 18.2% of the variance in P. The effects of measurement on hand vs arm and dominant vs non-dominant limb were insignificant. The main effect of a week of pregnancy was significant and negative, meaning that the more advanced the pregnancy, the lower the pain threshold (see Table 2). Specifically, each further week of the pregnancy decreased the pain threshold by 4.36 points. The main effect of physical activity was not significant, suggesting that the level of pain threshold was not associated with the current level of physical activity declared by participants. However, the main acute effect of the maximal exercise test was significant and negative, meaning that the pain threshold was lower by 23.48 points after the CPET than before the test (see Table 2). Moreover, the two-way interaction between physical activity and CPET was significant (see Table 2), indicating that the acute effect of the maximal exercise test on the pain threshold depended on the level of PA reported by participants.

**Table 2:** The results the multilevel regression analyses for PPT, PTOL and VAS score. Fixed.

predictor	df1	PPT			PTOL			VAS		
		df2	*F*	*p*	df2	*F*	*p*	df2	*F*	*p*
dominant limb vs nondominant limb	1	285.10	2.04	0.155	**284.2**	**16.4**	**< .001**	**282.95**	**10.58**	**0.001**
hand vs arm	1	285.10	0.00	0.992	**284.3**	**25.6**	**< .001**	283.01	0.84	0.360
WP	1	**304.17**	**52.90**	**< .001**	302.0	3.2	0.076	302.02	0.04	0.834
PA	2	249.45	0.94	0.394	**291.0**	**3.9**	**0.021**	**281.92**	**3.65**	**0.027**
Before CPET vs after CPET	1	**285.10**	**16.76**	**< .001**	284.3	2.8	0.097	282.98	1.25	0.264
PA X (Before CPET vs after CPET)	2	**285.11**	**3.12**	**0.046**	284.3	2.2	0.113	283.01	1.17	0.311
WP x (Before CPET vs after CPET)	1	285.10	1.66	0.198	284.3	0.2	0.663	283.02	0.60	0.439
WP x PA	2	267.91	1.40	0.248	**299.1**	**10.8**	**< .001**	294.29	0.42	0.661
WP x PA x (Before CPET vs after CPET)	2	285.11	0.88	0.416	284.3	0.3	0.763	283.07	1.68	0.187

*Note*. Satterthwaite method for degrees of freedom. PPT = pain threshold, PTOL = pain tolerance, VAS = subjective pain measured with Visual Analog Scale, WP = week of pregnancy, PA = level pf physical activity, CPET = maximal progressive cardiopulmonary exercise test. Values in bold represent statistically significant effects at *p* < 0.05

**Fig 2:**
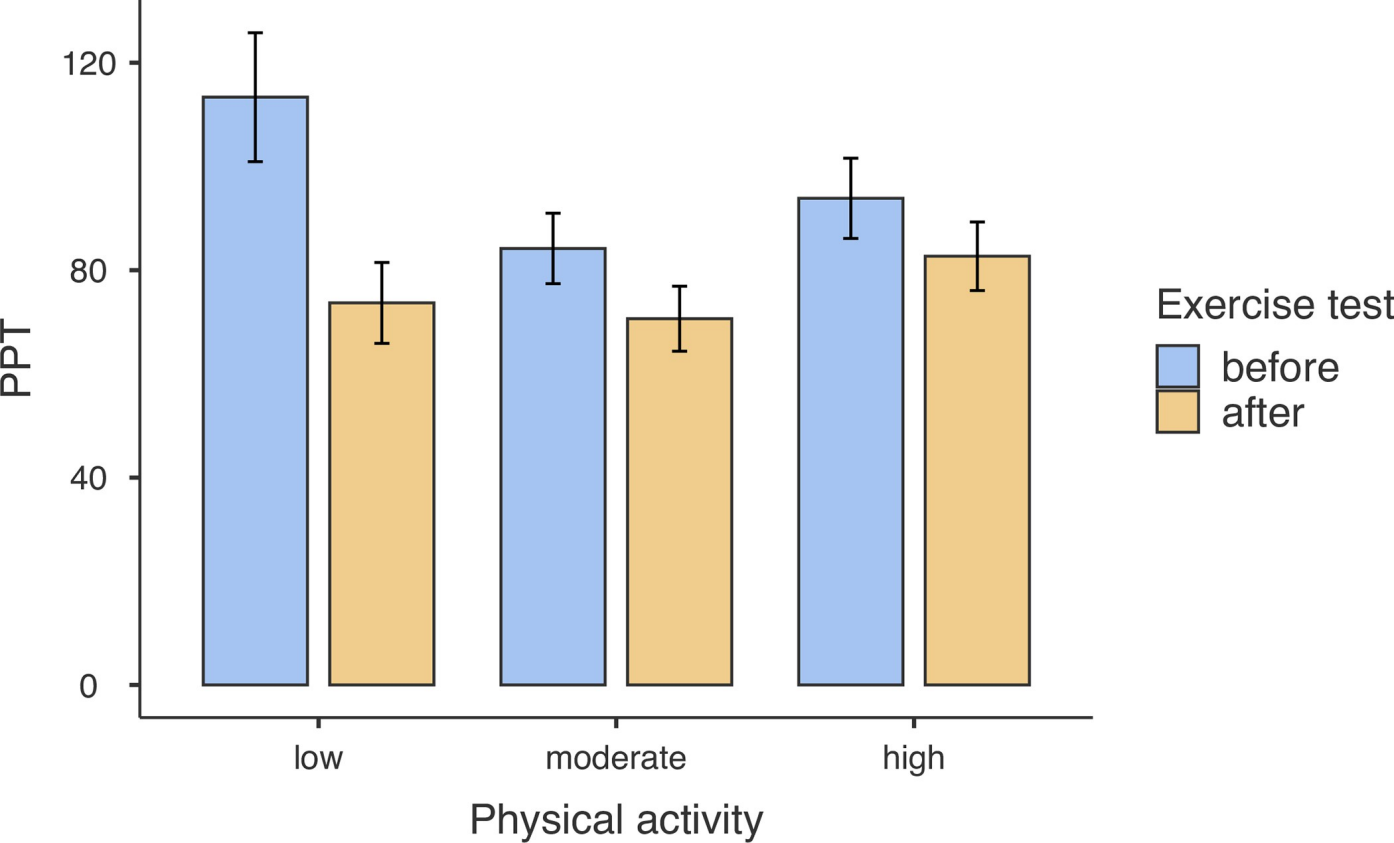
Pain threshold (PPT) as measured before and after the maximal progressive cardiopulmonary exercise test depending on the level of physical activity. Bars represent standard errors.

Further decomposition of the significant interaction between PA and CPET revealed that the acute effect of maximal exercise test was significant for participants with the lowest level of physical activity, b = -45.91, se = 12.00, 95%CI [-69.52, -22.29], t = -3.83, p < .001 but not significant for participants with medium, b = -13.73, se = 7.95, 95%CI [-29.38, -1.92], t = -1.73, p = .085, and high level of physical activity, b = -10.74, se = 9.94, 95%CI [-29.24, -1.92], t = -1.14, p = .254 (see Fig 2).

Finally, the two-way interactions with the week of pregnancy were insignificant, the same as the three-way interaction between the PA, CPET, and week of pregnancy, indicating that the abovementioned effects of PA and CPET were independent of the stage of pregnancy.

### Pain tolerance

The same analysis with pain tolerance as a dependent variable (see Table 2) demonstrated that the independent variables and covariates accounted for R^2^_marginal_ = 16.7% of the variance in PTOL. The effects of PTOL measurement on the dominant vs non-dominant limb were significant (see Table 2), such that pain tolerance was higher when measured on the dominant limb (*M* = 761.13, *SE* = 36.11, 95%CI [686.03, 836.23]) than non-dominant limb (*M* = 682.48, *SE* = 36.11, 95% CI [607.38, 757.58]). Also, the effects of PTOL measurement on hand vs arm were significant, such as pain tolerance was higher when measured on the hand (*M* = 771.11, SE = 36.09, 95%CI [696.05, 846.17]) than when measured on the arm (*M* = 672.50, SE = 36.14, 95%CI [597.35, 747.64]).

**Fig 3:**
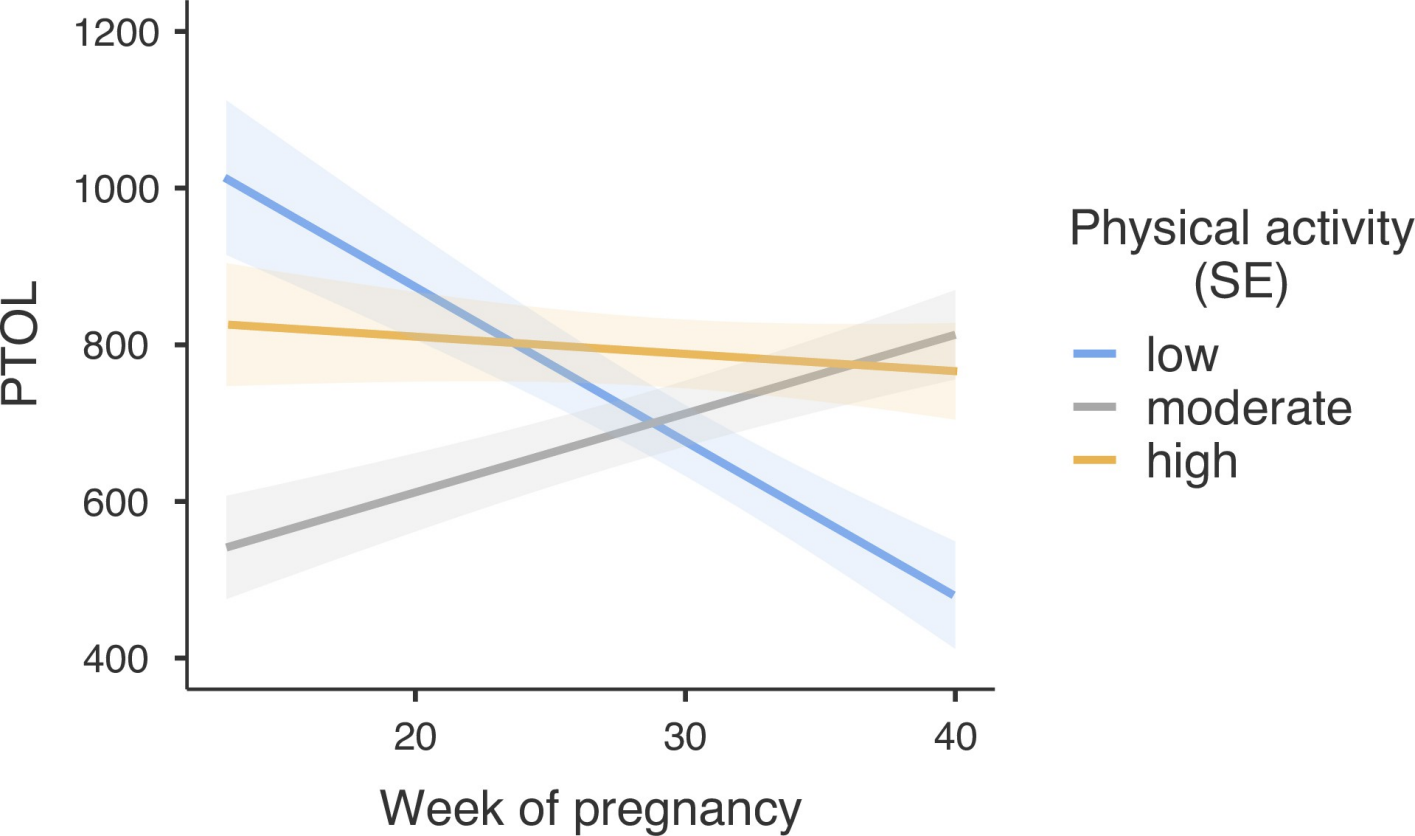
Pain tolerance (PTOL) depending on the level of physical activity and the week of pregnancy. Shaded areas represent standard errors.

The main effect of a week of pregnancy was insignificant, suggesting that, on average, pain tolerance did not change over the course of pregnancy. The main acute effect of the maximal exercise test was not significant, while the main effect of PA was significant. Further investigation of this main effect with post-hoc tests using Bonferroni corrections revealed that [1] women with low level of PA (*M* = 681.44, SE = 42.73, 95%CI [594.92, 767.97]) did not differ in terms of their pain tolerance from those of medium PA (*M* = 698.26, SE = 38.30, 95%CI [619.41, 777.12]), *t*(299) = -0.47, *p* > .999; but [2] women with a high level of physical activity (*M* = 785.71, *SE* = 40.87, 95%CI [702.38, 869.04]), on average, had pain tolerance score points higher than those with a medium level of PA, *t*(277) = -2.41, *p* = .049, and those with a low level of PA, *t*(299) = -2.69, *p* = .023. (See Fig 3).

The progressive cardiopulmonary exercise test (CPET) did not interact with the level of physical activity or the week of pregnancy, nor we found a three-way interaction between CPET, PA, and the week of pregnancy. However, a significant two-way interaction between the level of physical activity and the week of pregnancy was found (see Table 2). Further decomposition of this interaction (see Fig 2) revealed that among women with a low level of physical activity, the week of pregnancy was negatively associated with pain tolerance, *b* = -19.75, *SE* = 5.18, 95%CI [-29.95, -9.55], *t* = -3.81, *p* < .001, meaning that each further week of the pregnancy decreased the pain tolerance by 19.75 points. In turn, among women with a medium level of physical activity, the pain tolerance was positively associated with the week of pregnancy, *b* = 10.06, *SE* = 3.35, 95%CI [3.47, 16.65], *t* = 3.00, *p* < .001, meaning that each further week of the pregnancy increased the pain tolerance by 10.06 points. Finally, among the women with the highest level of physical activity, the pain tolerance was not associated with the week of pregnancy, *b* = -2.21, *SE* = 4.07, 95%CI [-10.21, 5.80], *t* = -0.54, *p* = .588, and remained relatively high through the pregnancy.

### Visual analog scale (VAS)

Regarding subjective pain perception, regression analysis showed that the independent variables and covariates accounted for R^2^_marginal_ = 6.7% of the variance in the dependent variable (see Table 2). A significant effect was observed for the dominant vs non-dominant limb, such that subjectively, the experience of pain was weaker in the dominant limb (*M* = 6.43, *SE* = 0.21, 95%CI [6.00, 6.87]) than in the nondominant limb (*M* = 6.79, *SE* = 0.21, 95%CI [6.36, 7.22]). The main effect of PA was also significant. Further investigation of this main effect with post-hoc tests using Bonferroni corrections revealed that [21] women with low PA did not differ in terms of their subjective level of pain (*M* = 6.65, *SE* = 0.25, 95%CI [6.15, 7.15]) from those of medium PA (*M* = 6.33, *SE* = 0.22, 95%CI [5.87, 6.78]), *t*(300.14) = 1.56, p = .358, and from those of high PA (*M* = 6.86, *SE* = 0.23, 95%CI [6.39, 7.34]), t(299.48) = -0.99, *p* = .977; but (2) in women with a high level of physical activity, on average, subjective pain experience was stronger than in those with a medium level of PA, *t*(265.57) = -2.62, *p* = .028.

## 4. Discussion

Exercise-induced analgesia is a topic studied in the context of various events, mostly related to competitive sports. The most consistent results on exercise-induced analgesia have come from studies in which electrical or pressure stimuli were used to applicate pain.

Given the subjective nature of pain, it is expected that less intense efforts undertaken by previously moderately active or physically inactive but highly positively motivated individuals such as pregnant women should trigger similar neurophysiological mechanisms. However, to our best knowledge, no studies investigated the effects of PA, including a maximal progressive exercise test, on pain sensitivity in pregnant women, measured as pain threshold and pain tolerance. This paper is to address this gap.

First, our results show that, on average, the pain threshold decreases during pregnancy, whereas pain tolerance and subjective pain perception remain at the same level from the second to the third trimester. Second, pregnant women in our study had significantly lower pain thresholds after a maximal exercise test than before. This effect was pronounced in women with low levels of PA, but it was observed regardless of whether the test was performed in the second or third trimester of pregnancy. The higher pain threshold after an exercise test might be related to the emotional state people experience after the end of intense physical exertion [28]. Third, we found that the PA level was significantly associated with pain tolerance, such that women with high PA had higher pain tolerance than women with moderate and low PA. In addition, physical activity levels predicted changes in pain tolerance over the course of pregnancy, with negative changes in low-PA women and positive changes in moderate-PA women. In turn, we observed no changes in pain tolerance in women with high PA, probably because their pain tolerance was already high at T1. Finally, these associations were not reflected in differences in the subjective pain perception.

Our results suggest that post-exercise analgesia may play an important role in pregnant women whose organisms are stressed by both the developing child and the future, inevitable labor pain. Labor pain has a significant impact on fear of childbirth (FOC), a common problem that affects women’s health and well-being during pregnancy and in the postpartum period and is a serious limitation of PA [29]. In addition, pregnant women often have misconceptions about the effects of exercise on fetal health or believe that PA during pregnancy can harm the fetus and cause miscarriage [17, 30–32]. This may be why women gradually reduce their PA during pregnancy [33] or limit it to half of the normal level [34]. In addition, studies have shown that pregnant women are significantly more likely to spend their time inactive (and choose a sedentary lifestyle) than non-pregnant women [35]. Studies conducted among thousands of women indicate that more than half of the women surveyed have limited knowledge about the effects of PA on pregnant women’s health. Most of this information comes from the Internet. In the study by Dudoniene et al. (2023) only 13.7% of all pregnant women surveyed knew about the role of physical therapy in back and low back pain. Therefore, educational programs, such as the one we relied on in this study, may be a solution to overcome such barriers [36]. The educational program implemented in our study addressed healthy lifestyles, but most importantly, the promotion of PA. Although 25% of our participants decreased their level of PA after 8 weeks of an online program, the remaining 75% either did not change it or even increased it. Therefore, it seems necessary to make pregnant women aware of the benefits of PA and encourage them to achieve and maintain high levels of PA during pregnancy.

Interestingly, our data show associations between subjective pain perception in response to a mechanical pain stimulus and the week of gestation or participation in the exercise test. The only differences were found in relation to the dominant vs non-dominant limb and across the different levels of PA. The former differences reflected differences in pain tolerance: participants could tolerate a higher pain level, and their subjective feeling of pain was lower on the dominant than on the non-dominant hand. These effects could be related to frequent exposure to stimuli and frequent use of the dominant vs non-dominant limb. The differences in subjective pain between women of different levels of PA are much smaller and more difficult to interpret, as the only significant difference was found between women with high PA (declaring highest subjective level of pain) and medium PA (declaring lowest subjective level of pain). Possibly, women with a high level of PA experienced subjectively more severe pain but were still able to tolerate it, as compared to women with lower levels of PA.

In summary, the effect described in the literature as exercise-induced analgesia is not a phenomenon that occurs only in competitive sports but is also possible in pregnant women not involved in such activities. Our study and data from the literature suggest that this subjective effect of a reduction in response to pain stimuli under the influence of individually chosen physical activity may be triggered by the interaction of several factors.

### Strengths and limitations of the study and implications for future research

One of the strengths of our study is the measurement of PPT and PTOL before and after the maximal CPET up to refusal, which gave a very strong physical stimulus for the pregnant body. The available scientific literature is dominated by studies in which pregnant women are subjected to submaximal CPET. The educational program is also valuable, as it combines not only content on physical activity, but also relaxation techniques, breathing exercises and birthing positions, as well as visualization of labour pain and coping with it. All these educational elements could have influenced the effectiveness of our intervention. However, to confirm this thesis, more detailed research should be carried out.

The first limitation of our work is the relatively small sample size and relatively high attrition. Although we did not find significant differences in demographic characteristics and most pain sensitivity variables between those who participated in the T2 measurements and those who did not, it is still possible that attrition is due to factors that were not controlled for in the study. Although we attempted to address the consequences of a small sample by using a mixed-subjects design in which each participant completed multiple measurements, our results may be underpowered. Second, we used a declared level of PA and therefore have no control over this variable and its reliability. Therefore, we see a need for future studies that are conducted with larger samples and use objective measures of physical activity, such as accelerometers. We also see a need for intervention studies with rigorous monitoring of women’s participation in PA sessions, ideally in a randomized design. Third, in this study, we only analyze data collected before and after the 8-week period of our intervention. It would therefore be interesting to examine changes in pain-related variables over a longer period, preferably throughout pregnancy. Finally, it would be particularly interesting to study a larger group of pregnant women just before delivery. Such an approach would allow us to determine the potential influence of birth hormones with the interaction of the acute effect of maximal physical exertion on changes in pain perception.

## Supporting information

S1 Checklist (DOCX)
